# A randomized, home-based, childhood obesity intervention delivered by patient navigators

**DOI:** 10.1186/s12889-015-1833-z

**Published:** 2015-05-23

**Authors:** Lourdes Yun, Richard E. Boles, Matthew A. Haemer, Shanna Knierim, L. Miriam Dickinson, Heather Mancinas, Simon J. Hambidge, Arthur J. Davidson

**Affiliations:** Denver Public Health, 605 Bannock Street, Denver, CO 80204 USA; Department of Pediatrics, University of Colorado Denver School of Medicine, 12631 East 17th Avenue, Aurora, CO 80045 USA; Denver Health Ambulatory Care Services, Department of Pediatrics, 777 Bannock Street, Denver, 80204 USA; Department of Family Medicine, University of Colorado Denver, 12631 E. 17th Avenue, Aurora, CO 80045 USA; Denver Health, Division of Ambulatory Care, 666 Bannock Street, Denver, 80204 USA

**Keywords:** Childhood obesity, Low-income, Latino, Patient navigator, Home-based obesity intervention, Preschool

## Abstract

**Background:**

Although Colorado is perceived as a healthy state, in 2010, 14.1 % of children aged 2–5 were overweight and 9.1 % were obese. Despite the high prevalence of obesity in this population, evidence to support particular strategies to treat obese preschoolers is lacking. The efficacy of home-based, childhood obesity interventions to reduce a child’s body mass index is inconclusive. However, this model uniquely provides an opportunity to observe and intervene with the home food and activity environment and engage the entire family in promoting changes that fit each family’s unique dynamics.

**Methods/design:**

Eligible participants are children aged 2–5 years who attended a well-child care visit at a Denver Health Community Health Service clinic within 12 months prior to recruitment and on that visit had a body mass index (BMI) >85th percentile-for-age. Participants are randomly recruited at study inception and allocated to the intervention in one of five defined 6-month stepped wedge engagements; the delayed intervention groups serves as control groups until the start of the intervention. The program is delivered by a patient navigator at the family’ home and consists of a 16-session curriculum focused on 1) parenting styles, 2) nutrition, and 3) physical activity. At each visit, a portion of curriculum is delivered to guide parents and children in selecting one goal for behavior change in each of three work areas to work on during the following week. The primary study outcome measure is change in BMI z-score from baseline to post-intervention period.

**Discussion:**

This childhood obesity study, innovative for its home-based intervention venue, provides rich data characterizing barriers and facilitators to healthy behavior change within the home. The study population is innovative as it is focused on preschool-aged, Latino children from low-income families; this population has not typically been targeted in obesity management assessments. The home-based intervention is linked to clinical care through update letters and assessment of the program’s impact to the child’s medical providers. Informing primary care providers about a child’s accomplishments and challenges, allows the clinician to support the health weight effort when seeing families during subsequent clinical visits.

**Trial registration:**

ClinicalTrials.gov NCT02024360 Registered December 21, 2013

## Background

Childhood obesity in the United States has reached an alarming prevalence rate, affecting all age groups including preschool children [1–3]. Obesity in children aged 2–5 years has increased from 5 % in 1971–1980 to 10.4 % by 2000 [2] to 12.1 % by 2009–2010 [4]. While several reports [5–7] describe improving obesity trends, Colorado rates continue to increase with the nation’s second highest rate of childhood obesity increase [7, 8]. In 2010, 14.1 % of Colorado’s children aged 2–5 years were overweight and 9.1 % were obese [9]. Poverty, an important social determinant of health, correlates significantly with obesity. In 2014, 18 % of children in Colorado lived in poverty, with Denver County having an even higher rate (29 %) [10].

Despite high preschooler obesity prevalence, evidence to support particular treatment strategies is lacking and convenient access to community-based treatment is limited [11]. While previous treatment studies often excluded children younger than 6 years of age [12], recent data suggest greater treatment success among preschool aged children than older children [13–16]. The US Preventive Services Task Force (USPTF) has recommended that primary care providers screen for and then refer obese children over 6 years of age to high quality treatment programs; no obesity screening recommendations were made for children younger than 6 years due to relatively limited treatment offered to this age group [17]. Testing treatment protocols for preschool children may build evidence for future screening recommendations for young children. Ideal protocols would be feasible, cost-effective, and reach low-income and minority children, who are at greatest risk for persistent obesity.

Research suggests that patient navigators and lay health workers can improve outcomes for chronic disease in adults [18–20] and children [21] and are effective in facilitating behavior change in various settings [22]. The efficacy of home-based, childhood obesity interventions to reduce children’s body mass index (BMI) is unknown [15, 23]. The home-based model is unique by providing an opportunity to observe and intervene with the home food and activity environment [13, 15, 23, 24]. In addition, family-based obesity interventions involving the child and the parent are associated with positive outcomes since families treated together share common treatment goals [25–27]. When the caregiver is included in obesity management programs [28, 29], families have demonstrated changed behaviors; intensive lifestyle and parenting skills education has resulted in sustained weight loss [30–33].

The Community Outreach Obesity Prevention Trial (COOPT) is an ongoing, 4-year (October 2011-September 2015) randomized controlled trial that tests the effectiveness of a home-based patient navigator program delivered to preschoolers of a large urban safety-net health care system. The intervention targets children aged 2–5 years within a predominantly Latino population. The primary goals are to examine the effect of the intervention in reducing the BMI z-score in the patient and the changes in health behaviors using the “5-2-1-0” daily targets (i.e., 5 fruits and vegetables, less than 2 hours screen time, 1 hour or more of exercise and 0 sweetened drinks) [34] using a home-based, patient navigator-mediated childhood obesity intervention.

COOPT is delivered by patient navigators at a family’s home. By focusing the program on the family unit, the parent sets goals for the child taking into account family dynamics around nutrition and physical activities. Primary care providers often lack the time to offer intensive weight counseling to families since the average well child visit is less than 20 minutes [35], and many providers do not feel they have the tools or knowledge to provide adequate weight management counseling [36, 37]. This intensive 25-hour program provides [38] a home-based platform for patient navigators to offer hands-on opportunities for skill-building, provide detailed education and counseling to families, reinforce learned concepts throughout the intervention, and offer opportunities for families to set, evaluate, and modify goals, as necessary.

## Methods/design

### Overview and hypotheses

**Fig. 1:**
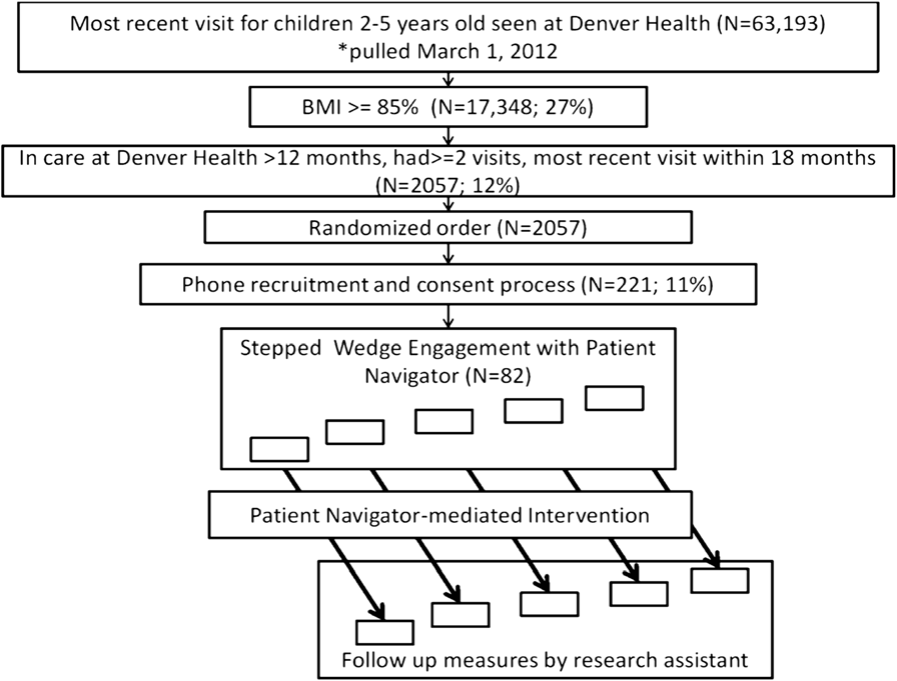
Flow of Children into the Study: Randomization, Recruitment, Enrollment, and Intervention Processes, Denver Health, 2012-2015

Overweight or obese children [9] aged 2–5 years (target *n* = 300) receiving medical care at one of eight community health clinics of an urban safety net medical system are randomly selected from a population of greater than 2000 eligible overweight or obese children to participate in this 16-session, home based, family-centered curriculum around nutrition, physical activity, and parenting skills. All participants are recruited at baseline and individual children and families are randomized to begin treatment in one of several sequential 25-hour stepped wedge cohorts. Children enrolled at inception but not receiving the intervention until a later start date are considered a control group for the period from recruitment until the intervention begins (Fig. 1).

The primary outcome measure is BMI z-score over time in children receiving the intervention compared to waitlist controls (delayed intervention). We hypothesize that children receiving the intervention will have a greater reduction in BMI z-scores than children in the waitlist control group. We will compare the change in BMI z-score slope of the two groups from baseline to follow-up period. The follow-up period of the intervention group is from the start through the end of the intervention. The follow-up period for the delayed intervention group is from recruitment to the start of the intervention.

The secondary outcome is change in health behaviors using the “5-2-1-0” daily targets (i.e., 5 fruits and vegetables, less than 2 hours screen time, 1 hour or more of exercise, and 0 sweetened drinks). We hypothesize that children receiving the intervention will experience a greater improvement in “5-2-1-0” measures from baseline to post-intervention compared with children in control group. A thorough evaluation of the delivery processes and acceptance by stakeholders will inform the potential for implementation and dissemination of the intervention.

### Study setting and participants

Denver Health [39] (DH), an integrated urban safety net health system, includes a 477-bed hospital, 16 school-based clinics, and eight federally qualified community health centers. DH provides services to 25% of Denver residents, 35% of Denver’s children, and a large proportion of indigent and minority populations (58% Latino, 21% Caucasian, 17% African American; 4% other races). Among DH primary care patients, 98% are below 200% of the federal poverty guidelines and most (87%) are publicly insured or uninsured patients. In 2013, among 42,521 children 2–18 years old seen at DH, 32% (13,944) were overweight or obese; of whom, nearly 11,000 (79%) were Hispanic or Latino.

Eligible participants for the study are children aged 2 to 5 years who have been receiving well-child care at a Denver Health Community Health Service clinic for at least 12 months, had two or more visits with the most recent visit within 18 months, and have a BMI >85th percentile-for-age, recorded at least 9 months prior to the effort to recruit. Children with a physical or developmental disability/condition that precludes measurement of standing height, or a chronic (i.e., greater than 1 month) physical or developmental disability/condition that precludes age-appropriate participation in routine physical activity are excluded.

This study was approved by the Colorado Multiple Institutional Review Board (Protocol 11–1700).

### Recruitment and randomization

Participant recruitment is performed from a randomized list of overweight or obese patients who had a BMI measure recorded at least 9 months prior to study initiation (Fig. 1). Biologically implausible values for height or weight are excluded using a CDC-developed algorithm [40]. A research assistant contacts potential enrollees from the randomized list. Prior to the call, families are mailed a postcard with a brief description of the program and informed that they will soon be contacted. Once called, a bilingual English-Spanish research assistant explains the study to the family over the phone. If the family agrees to participate, the research assistant reads a verbal consent and documents consent. After oral consent is obtained, a baseline set of behavioral questions is administered. The survey is re-administered if more than 30 days have lapsed between the administration of the baseline survey and the start of the intervention. These baseline pre-intervention behaviors are compared with post-intervention and 6-month post-intervention behaviors recorded on subsequent questionnaires.

Study participants are told they will be scheduled to begin patient navigator visits in a staggered manner, using a stepped-wedge design [41] with individuals assigned to an intervention cohort to start the intervention. Those not enrolled in the initial intervention cohort are sent quarterly newsletters with information and resources unrelated to the intervention to keep participants connected to the study, during the delayed period.

### Intervention

At the beginning of the intervention, the patient navigator contacts a cohort of previously recruited families to schedule the first home visit. The first home visit is aimed at creating a positive rapport between the caregiver and the patient navigator and to set program expectations and goals. At this visit, the patient navigator sets lifestyle change and weight goals with families. The following talking points are used to explain the program’s goals to families: 1) program goal is to help your preschooler grow at a healthy rate which means your child’s weight will be matched well for height; 2) pre-school children who carry too much weight for good health can gradually become healthier by slowing the rate of weight gain and then holding their weight steady; 3) as your child grows taller the weight will better match the height and the risk of illnesses like diabetes and heart disease are expected to be lower than if the child remains obese into adulthood; 4) rarely should we expect preschoolers to lose weight, if they do, losing no more than one pound per month can be healthy and weight loss is usually not needed unless preschoolers are severely overweight; and 5) the best way to help preschoolers grow healthier is to practice healthy eating and exercise habits as a whole family. At this initial visit, the patient navigator administers a written consent, and a copy of the consent forms are provided to each family.

At each subsequent home visit, the patient navigator delivers a portion of the curriculum focused on parenting styles, nutrition, and/or physical activity to guide parents and children in selecting one goal for behavior change in each of the three areas to work on the following week. Families report progress towards these goals at the following session. Patient navigators exercise flexibility to change the order of the sessions, spending more or less time on a topic tailored to the family’s level of understanding or mastery of skills. The full curriculum content is delivered regardless of the order of the sessions. Home visitation sessions usually occur on weekdays before 7:30 pm; occasionally the visits occur on Saturdays to accommodate the families’ schedules. The entire family, including siblings 3–18 years of the index overweight or obese child, is invited to participate in the sessions.

A nurse-practitioner, leader of clinical obesity services for the Denver Health Community Health Services, is liaison to facilitate interactions between patient navigators and clinicians. Health care providers of the children participating in the program receive three letters throughout the program: 1) at enrollment, informing that the child started the program, 2) at mid-intervention, listing the child’s progress and goals, and 3) at completion, summarizing the child’s accomplishments, future goals and challenges in achieve current goals. These letters inform the provider during subsequent clinical visits to engage in discussion to support the family’s achieving goals set with the patient navigators during the program.

### Curriculum

The curriculum used in this program was extensively adapted from a family-centered, telephone-delivered, childhood obesity treatment program developed by Kaiser Permanente “Family Connections”[42]. The curriculum was roughly doubled in intensity and translated into 16 home/community visits that occur over a 25-hour period. The choice of curriculum components and mode of delivery was purposeful. To ensure cross-cultural acceptance of the intervention, the American Diabetes Association Latino Outreach Program was consulted during program adaptation. With assistance from the Latino Outreach Program, two focus groups with mothers and grandmothers of preschool aged-children were conducted. Based on feedback from the focus groups, curriculum tracks on parenting skills, helping preschoolers accept healthy foods, and hands-on cooking and shopping skills were added to the “Family Connections” curriculum by clinical experts who are part of the COOPT program.

The program curriculum focuses on nutrition, physical activities, and parenting skills delivered in the participant’s home (Table 1). Topics include parenting styles (e.g. limit setting, conflict resolution) and skills for facilitating healthy behavior change (e.g. goal setting, troubleshooting, positive reinforcement) for parents of preschoolers. Nutrition education focuses on selecting healthy foods and healthy portions for the entire family. During family visits children participate in discussions designed to educate them about healthy foods (i.e., fruits, vegetables, whole grains, and lean proteins), at their developmental level.

**Table 1:** Community Outreach Obesity Prevention Trial: Curriculum learning objectives

Session no.	Theme	Learning objectives:
1	Develop Rapport, Baseline Measurement; Explain 5-2-1-0 and Track Behaviors	1. Create rapport between family and navigator
		2. Family will be able to explain the study process and purpose
		3. Collect Baseline Data (Baseline measures)
		4. Explain 5-2-1-0 concepts
		5. Describe reasons for children’s growth patterns
		6. Describe your own family health behaviors
		7. Demonstrate how to keep track of behaviors and set goals
2	Nutrition: Focusing on family meals	8. Explain the structure for visits
		9. Explain 5-2-1-0 : focus on 5
		10. Describe 2 strategies for improving fruit and vegetable consumption
		11. Demonstrate rule setting around eating and food choices
		12. List a specific family challenge
		13. Describe how to keep track of behaviors and set goals
3	Physical Activity: Introduction	14. Explain “5-2-1-0”: 1 hour or more of physical activity every day
		15. Explain “5-2-1-0”: 2 hours or less recreational screen time per day
4	Being in charge and making changes	16. Describe a parent’s role during meal times
		17. Explain concept of food neophobia
		18. Describe use of differential attention (praising/ignoring)
5	Being a good judge of home health	19. Explain “5-2-1-0”: zero sugary drinks every day
		20. Explain why a home health assessment is valuable
		21. Compare PN and parent home health assessment results
6	Being focused on choices	22. Explain how to read food labels
		23. Describe whole grains, fiber
		24. Compare nutritional value of selected foods
		25. Explain what lean protein means and what foods contain them
		26. Describe low-fat dairy products and how to include in your meal planning
7	Grocery Store Outing	27. Demonstrate reading food labels
		28. Demonstrate comparison shopping and calculate unit pricing
		29. Describe ways to buy fruits and vegetables on a limited budget
8	Cooking Demonstration	30. Describe the method of preparing a new food or recipe(s)
		31. List beneficial factors from new recipe(s)
		32. Compare this recipes nutritional value to a similar but less nutritious form
		33. Demonstrate kitchen skills required to prepare the recipe(s)
		34. Plan a meal considering food preferences
9	Identifying barriers and Staying Motivated	35. Demonstrate exercising while delivering other objectives (walk during session)
		36. Describe the common barriers to regular exercise
		37. Describe the process of building motivation for physical activity
10	Creating a healthy home and effective directions	38. Describe how to set a home for success, eliminating food with little nutritious value, putting foods out of site
		39. Explain how to give effective directions to your child
11	Family Support: Promoting healthy body image and discipline	40. Explain how we talk promotes and affects a healthy body image
		41. Describe the use of time out and other discipline techniques
12	Nutrition: Fruits, Vegetables and Portion Control	42. Describe the importance of promoting fruits and vegetables with every meal
		43. Explain the value of snacks and nutritional goals
		44. Explain how to prepare healthy affordable meals
		45. Demonstrate ability to prepare healthy affordable meals
13	Physical Activity: Overcoming Burnout or Motivating Self or PA	46. Explain what burnout means and how it can be avoided
		47. Explain the importance of dealing with changing schedules
		48. Describe ways to maintain focus but be flexible
14	Family Support: Finding support and putting it all together	49. List ways to include other family and friends
		50. Demonstrate ability to blend all learned skills
15	Family Support: Planning Ahead and Problem Solving	51. Describe the home change experience
		52. Explain efforts for home change
		53. Describe methods to plan ahead for challenges
		54. Describe problem solving skills and their use
16	Final Measures	55. Celebrate Success, Allow for Review and Reflection

To supplement the curriculum with pre-school specific physical content and activities, the SPARK curriculum and activity set [43] was purchased for use by the patient navigators.

The curriculum also includes 1) a grocery store tour in which the patient navigator guides a family in selecting low-cost healthy foods and 2) a cooking session when the family and the patient navigator prepare a dish that is enjoyed by the family using healthy ingredients. Families receive a 10 dollar grocery gift card for the grocery store tour.

The curriculum is delivered to each family by one consistent bilingual patient navigator in the parent’s preferred language (English or Spanish).

### Patient navigator training

The program employs two full-time bilingual English-Spanish patient navigators. Patient navigators receive extensive training shortly after being hired. A four-day course sponsored by the NIH-funded, Colorado Clinical Translational Sciences Institute provides Patient Navigator Fundamentals [44] that includes patient communication, health promotion, professional conduct and motivational interviewing. The course is designed to equip individuals with the knowledge and skills necessary to function as a patient navigator. Specific childhood obesity knowledge is achieved by attending and observing a childhood obesity program curriculum (similar to the one offered through this program) yet delivered to groups in a community recreation center setting; patient navigators also have direct one-on-one training with experienced clinicians and patient navigators. Additional training components include in-home observations of patient navigators and debriefing and evaluation sessions with an experienced [45–47] patient navigator supervisor.

### Outcome measures

#### Child measures

##### Height and weight

The primary outcome of the study is the child’s BMI z- score over time, used to assess change in BMI z-score from baseline to follow-up. Height and weight are measured at the first and last home visits. Standing height is measured with the child barefoot. Patient navigators are taught measurement techniques by experienced medical assistants in community pediatric clinics, overseen by study team nurse practitioner. To ensure accurate measurement in each session, height is measured three times and the average value is recorded. Children are weighed in light clothing, without shoes. Weight and height are measured on a LifeSource™ UC-321 Precision Scale and Charder HM200P Portstad Stadiometer. BMI percentile and BMI z-scores are calculated using age and sex-specific information based on the 2000 Center for Disease Control (CDC) growth charts [40].

##### Diet and physical activity

The physical activity and eating behaviors of the child as summarized in “5-2-1-0” daily targets are captured by a standard survey [48] administered to the child’s caregiver at the time of recruitment, at the completion of the intervention and at 6-months post-intervention. Families receive a 10 dollar gift card for each completed survey.

#### Environmental measures

To assess the home presence and availability of 20 fruits, 14 vegetables, 11 physical activity devices, and electronics in bedrooms (e.g., televisions), a home health assessment survey [49] is administered by the patient navigator at an initial (session 5) and a follow-up (session 15) home visits. Data from these surveys are used to compare changes in the home environment associated with the intervention received by families.

### Clinical provider measure

#### Process evaluation

At the end of the intervention period, a survey will be sent to health care providers of children participating in the program. The survey will collect provider perspectives of the program and value of receiving letters listing the child’s progress, goals/objectives: 1) increased knowledge of patient’s program participation, 2) helpful or unnecessary communication, and 3) affected the patient’s weight management care in the clinical setting.

#### Patient navigator measure

##### Process evaluation

To evaluate the experience and lessons learned from patient navigators working with families, we will analyze the patient navigators’ field notes and conduct individual interview with currents and available former patient navigators.

#### Family measure

##### Process evaluation

At the end of the intervention period, focus groups will be conducted with family caregivers graduating from the program to evaluate their perceptions, program usefulness and suggestions for improvement in curriculum content and delivery.

#### Intervention fidelity

At the end of each home visit, to monitor and assure fidelity to project goals and content, patient navigators use a Likert scale to assess whether learning objectives were: 1) delivered; 2) understood; 3) family’s perceived importance of the topic; and 4) family’s confidence in making a change. Patient navigator intervention assessments are recorded on paper and then transferred to a software application. To ensure the curriculum is delivered as intended, the patient navigators record the duration of each session by noting the start and end time of each session.

### Enrollment and retention efforts

Assistance from the child’s primary care provider is sought to improve program enrollment and retention. For families who decline to participate despite previously agreeing, or families who drop-out of the program after attending some sessions, the clinician liaison contacts the child’s provider and asks for permission to send a language-concordant letter to the child’s caregiver, on behalf of the clinician, encouraging program participation or completion.

### Sample size and power

The primary outcome is BMI z-scores over time from baseline to post-intervention in treated children, and from baseline to the start of intervention in delayed intervention children. We hypothesize that compared with children receiving delayed intervention, children in the intervention group will experience greater reduction in BMI z-scores over time as shown by a greater decrease in BMI z-score slope. A sample size of 80 intervention children and a frequency matched cohort (by age category) of 160 controls will provide 81% power to detect a 0.39 effect size difference between groups.

### Statistical analysis

#### Primary analysis

To determine the effect of the program on the BMI z-score of treated children, we will compare change in BMI z-scores for treated children to change in BMI z-scores in the delayed intervention control group over an equivalent time frame using general linear mixed effects models (growth curve models) with random intercept and random slopes, adjusting for patient and family-level covariates. Covariates that differ at baseline between the intervention and delayed-control groups will be included in multivariable models.

#### Secondary analysis

To determine intervention effect on patient physical activity and eating behaviors, we will compare the mean “5-2-1-0” index from baseline to post-intervention using the paired *t*-test. Additional analyses will be carried out using general linear mixed models with repeated measures on children to adjust for covariates and explore possible baseline predictors of change.

## Discussion

The need for robust childhood obesity interventions is evident for preschoolers, but limited current availability and capacity for comprehensive childhood obesity management mean many children have unmet service needs. Primary care providers ideally would provide counseling for families with overweight and obese children, but clinicians have: 1) competing demands, 2) limited time, knowledge and resources, and 3) lack of in-depth nutrition and physical activities counseling skills. Culturally competent patient navigators trained in supporting lifestyle change may be an effective adjunct to clinically-based obesity management services for preschoolers. They provide in-depth counseling around nutrition and physical activities to families.

**Table 2:** Environmental observations to assess in-home family chaos [50]

Environmental observation	Yes	No
TV on		
Music on		
More than one person talking at a time		
Cluttered rooms, making it difficult to walk around or meet for home visit		
More than family present in the home		
Non-compliance to parent directions (ignored or refused by child)		
Disrespectful language		
Inappropriate behavior between 2 people		
Answering the phone (texting) during visit		

Our study, The Community Outreach Obesity Prevention Trial (COOPT) is a randomized controlled trial testing a family-centered obesity treatment in a predominantly Latino population of preschool age children. This childhood obesity management program is delivered by patient navigators in a home setting. Patient navigators have a unique opportunity to develop a trusting relationship with families over a detailed 16-session curriculum around nutrition, physical exercises, and parenting skills, to assess the home food/exercise device environment, and to offer healthy recommendations for the families.

The study targets a low-income population who often experience social and environmental obstacles precluding a caregiver’s ability to make healthy changes for their child. For families with limited budget, the patient navigators work with the families to choose alternative healthy and affordable foods. When family and environmental chaos (Table 2 [50]) is observed during home visits, the patient navigators have the opportunity to address them with the families or to provide resources to assist the families. At the end of each session, the patient navigators assess the parent’s level of understanding of the session, the importance of the topic, and their confidence in making the proposed changes. At three points during the program (i.e., first session, mid-intervention, and last session), patient navigators assess parental readiness for change. Assessments at the end of each session, in conjunction with parental readiness to change, guide patient navigators to develop goals and objectives tailored to individual families’ needs and more likely to be achieved.

The study is innovative in its focus on preschool-aged, Latino children from low-income families; this population has not been typically targeted in obesity management programs. The study is also novel in the home-based intervention venue, which provides rich data about acceptance and characterization of barriers and facilitators of healthy change within the home. The home-based intervention links to clinical care through update/outcome letters and program impact assessments shared with medical providers. By informing primary care providers about a child’s program accomplishments and challenges, the clinician may personalize messages with families during subsequent clinical visits.

## Abbreviations

BMI: Body Mass Index
COOPT: Community Outreach Obesity Prevention Trial
USPTF: US Preventive Services Task Force
